# Fortified Balanced Energy-Protein Supplements Increase Nutrient Adequacy without Displacing Food Intake in Pregnant Women in Rural Burkina Faso

**DOI:** 10.1093/jn/nxab289

**Published:** 2021-09-07

**Authors:** Brenda de Kok, Alemayehu Argaw, Giles Hanley-Cook, Laeticia Celine Toe, Moctar Ouédraogo, Trenton Dailey-Chwalibóg, Loty Diop, Elodie Becquey, Patrick Kolsteren, Carl Lachat, Lieven Huybregts

**Affiliations:** Department of Food Technology, Safety, and Health, Faculty of Bioscience Engineering, Ghent University, Ghent, Belgium; Department of Food Technology, Safety, and Health, Faculty of Bioscience Engineering, Ghent University, Ghent, Belgium; Department of Food Technology, Safety, and Health, Faculty of Bioscience Engineering, Ghent University, Ghent, Belgium; Department of Food Technology, Safety, and Health, Faculty of Bioscience Engineering, Ghent University, Ghent, Belgium; Institut de Recherche en Sciences de la Santé (IRSS), Unité Nutrition et Maladies Métaboliques, Bobo‐Dioulasso, Burkina Faso; AFRICSanté, Bobo‐Dioulasso, Burkina Faso; Department of Food Technology, Safety, and Health, Faculty of Bioscience Engineering, Ghent University, Ghent, Belgium; International Food Policy Research Institute (IFPRI), Washington, DC, USA; International Food Policy Research Institute (IFPRI), Washington, DC, USA; Department of Food Technology, Safety, and Health, Faculty of Bioscience Engineering, Ghent University, Ghent, Belgium; Department of Food Technology, Safety, and Health, Faculty of Bioscience Engineering, Ghent University, Ghent, Belgium; Department of Food Technology, Safety, and Health, Faculty of Bioscience Engineering, Ghent University, Ghent, Belgium; International Food Policy Research Institute (IFPRI), Washington, DC, USA

**Keywords:** pregnant women, dietary assessment, 24-h recall, balanced energy-protein supplements, lipid-based nutrient supplements, displacement, nutrient adequacy, Burkina Faso

## Abstract

**Background:**

In many low- and middle-income countries, the prevalence of energy and nutrient deficiencies is high among pregnant women. Balanced energy-protein (BEP) supplements are a promising strategy to cover nutritional requirements during pregnancy and improve birth outcomes. However, the displacement of nutrient-dense foods by BEP might attenuate the efficacy of supplementation.

**Objective:**

This cross-sectional study of participants in a randomized controlled trial evaluated the difference in energy and macro- and micronutrient intakes, food groups, and nutrient adequacy between a control and intervention group receiving either a daily iron–folic acid (IFA) tablet or IFA and BEP supplement during pregnancy, respectively.

**Methods:**

We collected a single multiple-pass 24-h recall from 470 pregnant women from the MIcronutriments pour la SAnté de la Mère et de l'Enfant (MISAME) III study that investigates the efficacy of BEP supplementation on birth outcomes and infant growth. Dietary intake (median and IQR) and nutrient adequacy were assessed using individual recipes and preparation methods of mixed dishes for each participant. Linear regression models were fitted to compare energy and nutrient intakes.

**Results:**

Dietary energy, and macro- and micronutrient intakes were significantly higher among women in the intervention group when including BEP [2329 kcal/d (1855, 3008 kcal/d) compared with 1942 kcal/d (1575, 2405 kcal/d) in the control group (all *P* < 0.001)]. The difference in median energy intake (448 kcal/d; 95% CI: 291, 605 kcal/d) was approximately equivalent to a daily dose of the BEP supplement (393 kcal). Nutrient adequacy ratios for both groups were low for all micronutrients (between 0.02 and 0.66), when excluding BEP (except iron and folic acid, due to standard supplemental doses) from analysis. However, nutrient intakes increased to the Estimated Average Requirement for pregnant women when including BEP supplements.

**Conclusions:**

BEP supplementation increases energy and macro- and micronutrient intakes among pregnant women and fills nutrient gaps without displacing food intake. This trial was registered at clinicaltrials.gov as NCT03533712 (https://clinicaltrials.gov/ct2/show/NCT03533712).

## Introduction

During pregnancy, women have increased physiological needs for energy and nutrients to support fetal growth and development (1). The average total cost of pregnancy is estimated to be 90 kcal, 290 kcal, and 465 kcal/d for the first, second, and third trimester, respectively (2). In addition to energy, protein, and essential fatty acids, B-complex vitamins, vitamin A, vitamin D, iron, and zinc are needed to regulate fundamental processes of fetal growth, and iodine is particularly important for brain development (3). Optimal maternal nutrient intake is also imperative to maintain maternal metabolism and support maternal tissue growth to ensure adequate lactation performance for the newborn (4). Hence, inadequate diets among pregnant women can lead to adverse health outcomes in both the mother and child (5).

In many low- and middle-income countries, and in particular in food-insecure settings, the prevalence of micronutrient deficiencies is high, due to monotonous, nutrient-poor diets coupled with low bioavailability and poor absorption due to infections (3, 6, 7). Single micronutrient deficiency estimates among pregnant women range from 20% to 30% worldwide (4) and up to 40% in Africa (3). In Burkina Faso, a previous study has indicated that pregnant women do not meet any of the WHO/FAO-recommended daily nutrient allowances, except for phosphorus (8). Other studies have reported similar findings in Burkinabe mothers with young children (9, 10), women of reproductive age (11), and women living in urban areas (12). Furthermore, micronutrient needs of women cannot be met by a diet containing only commonly consumed local foods in Burkina Faso (13). Nutrition supplements are thus advised to fill micronutrient gaps on a short-term basis, and possibly represent a lower cost compared with nutrient-dense foods like animal-source foods. The following micronutrients were often found to be deficient by previous dietary intake studies in Burkina Faso: riboflavin, vitamin B-6, vitamin B-12, folate, calcium, and iron (8–13). The most recent reviews suggested that maternal multiple-micronutrient (MMN) supplementation and supplements containing protein, energy, and other nutrients (14–16) demonstrated a positive effect on several birth outcomes. Based on the available scientific evidence, the current WHO guidelines (17) therefore recommend providing nutritional supplements to cover nutritional requirements during pregnancy and lactation in undernourished populations.

Balanced energy-protein (BEP) supplements provide <25% of total caloric content from protein and have the potential to improve maternal nutritional status and fetal growth (18–21) in undernourished populations, yet large trials are required to support the current evidence for future recommendations and decision making. The MIcronutriments pour la SAnté de la Mère et de l'Enfant (MISAME) III study is an ongoing randomized controlled trial (RCT) assessing the efficacy of BEP supplementation on birth outcomes and infant growth. One important factor that may attenuate the efficacy of fortified BEP supplements is their displacement of (nutrient-dense) foods that are part of the base diet (22, 23). Therefore, the primary aim of this study was to assess whether a prenatal BEP supplement displaces part of the usual diet of pregnant women. A secondary aim was to assess the nutrient adequacy of pregnant women's diets with and without supplements.

## Methods

Our research was reported using the STROBE-nut (STrengthening the Reporting of OBservational Studies in Epidemiology–nutritional epidemiology) checklist (24).

### Main trial

Details of the MISAME-III efficacy trial are described elsewhere (25). In brief, 1776 pregnant women were individually randomly assigned to pre- and postnatal intervention or control groups. The intervention groups received a daily fortified BEP supplement and iron–folic acid (IFA) tablet and the control groups received an IFA tablet alone. In a formative study, the most preferred and suitable BEP was selected for administration in the RCT (26, 27). The selected BEP supplement belongs to the family of lipid-based nutrient supplements (LNSs) and consists of a peanut paste spread fortified with MMNs (**Table 1**). The advantage of this type of supplement is that it is an energy-dense, ready-to-consume product that does not require a cold chain, is highly stable, and has a long shelf-life. Anthropometric and sociodemographic data were collected at baseline. The study was approved by the Ethics Committee of Ghent University Hospital (B670201734334) and the Burkinabe Ethics Committee (no. 2018–22/MS/SG/CM/CEI) and written informed consent was obtained before enrollment. The study is registered at clinicaltrials.gov under identifier: NCT03533712.

**TABLE 1:** Nutritional values of the balanced energy-protein supplement

Ready-to-use supplementary food for pregnant and lactating women^1^	Mean for 72 g (serving size)
Total energy, kcal	393
Lipids, g	26
Proteins, g	14.5
Carbohydrates, g	23.3
Calcium, mg	500
Iron, mg	22
Zinc, mg	15
Vitamin A,^2^ μg RE	770
Thiamin, mg	1.4
Riboflavin, mg	1.4
Niacin, mg	15
Vitamin B-6, mg	1.9
Folic acid, μg	400
Vitamin B-12, mg	2.6
Vitamin C, mg	100

1 Ingredients: vegetable oils (rapeseed, palm, soy in varying proportions), defatted soy flour, skimmed milk powder, peanuts, sugar, maltodextrin, soy protein isolate, vitamin and mineral complex, stabilizer (fully hydrogenated vegetable fat, mono- and diglycerides).

2 1 μg vitamin A retinol equivalent (RE) = 3.333 IU vitamin A.

### Study population and recruitment

The study was conducted in 6 rural health center catchment areas located in the Houndé health district in the Hauts-Bassins region of Burkina Faso. Data were collected from September to October 2020, at the end of the preharvest season. According to a previous study conducted in the same area, the mean energy intake of pregnant women was 2050 kcal/d with an SD of 623 kcal (28). Based on that, a sample size of 234 subjects per study arm (468 in total) was required to detect a difference in energy intake of 196.5 kcal between arms, which corresponds to half the dose provided by the fortified BEP supplement, with *α* = 0.05, *β* = 0.90, and 10% nonresponse. We randomly selected 595 pregnant women enrolled in the MISAME-III trial with a gestational age between 12 and 34 wk, stratified per health center and intervention group, using the *runiform* function in Stata (StataCorp). The principal sample list consisted of 469 women. A back-up list of 126 women was generated in the event that women from the principal list were not available for an interview.

### Dietary assessment

Dietary intake was assessed by means of a single 24-h recall (24HR), conducted using a tablet-based multiple pass interactive strategy (29) implemented in SurveySolutions software (version 20.10; World Bank Group). Six enumerators and 1 supervisor with experience in 24-h dietary recall assessment from other studies were recruited for this study. The field team received a 1-wk refresher training in September 2020 on interviewing techniques, portion-size estimation, and data entry on tablets. After the training, 2 d were scheduled for pilot testing the recall method with pregnant women not enrolled in the study.

Two days before the dietary assessment, standard plates and bowls were provided to the women to be used to serve their own food, instead of eating with other family members from a shared plate or individual plates that differ in size, which would hamper harmonized individual portion-size estimation. The procedure was explained to the participants and it was emphasized not to change their eating habits in the interest of the study. Recall days were evenly distributed over the entire week, from Monday to Sunday.

The 24HR method consisted of 5 passes during which each woman was asked to recall all foods and drinks she consumed between waking up the previous day until waking up on the day of the recall. In the first pass, the woman was asked to report all foods and drinks consumed. In the second pass, additional details about the foods and drinks she listed were collected, including time of consumption, preparation method (raw, boiled, grilled, etc.), and a detailed food name. In the third pass, details of mixed dishes were collected: a list of ingredients with its form (fresh, dry, etc.) and quantity, any leftovers, preparation method of the mixed dish (boiled, fried, etc.), and total volume of the prepared mixed dish. In the fourth pass, she was asked to estimate the portion size and any leftover of each food or drink consumed. Different prespecified portion-size estimation methods were used: actual foods and salted replicas for main staples (e.g., *tô*, a stiff cereal porridge, and rice), playdough for pieces of food (e.g., meat, fish, snacks), volumetric measures for liquids (e.g., sauces, thin porridges, beverages), household measures using project utensils, photos using an atlas with common foods (e.g., vegetables, fruit, fish, and mixed dishes), and prices (e.g., bread). A digital scale with 1-g precision was used. In the fifth pass, the enumerator reviewed the information recorded in the previous passes and checked for any missing foods. The 24HR also included questions on the consumption of BEP supplements, IFA tablets, or other supplements and qualitatively assessed whether the reported quantity and variety of intake were habitual compared with the woman's base diet.

### Data preparation

A list of commonly consumed food and drinks in Burkina Faso, which were enumerated during 2 previous 24HR studies conducted by the Soutenir l'Exploitation familiale pour Lancer l’Élevage des Volailles et Valoriser l’Économie Rurale (SELEVER) trial (30), was preloaded into the questionnaire and could be directly selected from a drop-down menu, each with a unique code. New foods could be added to the questionnaire throughout the study whenever needed.

Portion sizes measured by different methods were converted to edible portions into grams using conversion factors (food density or per-unit measures) from context-specific conversion lists from the SELEVER trial (30), or collected by the supervisor after the study if the conversion factor was not available in this database. For mixed dishes, we calculated the amount of each ingredient consumed using the amount of raw ingredients used for preparation together with the total amount of the prepared dish and the amount consumed by the participant. For the few cases where a household recipe was not reported, average recipe values from our study population were used or, if these were absent, standard recipes values from the West African food-composition table (FCT) were used (31).

Energy and nutrient intakes were calculated using food-composition data from the West African FCT (31) with additional data from another FCT adapted to Burkinabe foods (32). In case of missing data, values of similar food items in the West African FCT were used. This was mainly the case for a few micronutrients of local green leaves (e.g., Moringa) for which values from the food code “mixed/unknown green leaves” were used. For the shea tree caterpillar (*Cirina butyrospermi*) that was consumed by 12 participants, available mineral values from the literature were used (33). Nutrient values for raw ingredients of recipes that were either boiled, steamed, grilled, or fried were adjusted for nutrient losses using retention factors (34). Because the BEP was peanut-based, we specifically looked into the intake of peanuts and peanut-based products. Finally, to estimate the nutrient adequacy of the diet, Nutrient Adequacy Ratios (NARs) were calculated by dividing nutrient intakes by the Estimated Average Requirements (EARs) for pregnant women by age group, truncated at 1 (35). An NAR equal to 0 indicates a diet that does not meet the requirements, whereas an NAR equal to 1 indicates a diet that achieved or exceeded the recommended nutrient intake for that nutrient. For iron, we used recommendations adjusted for low (5%), medium (10%), and high (16%) bioavailability (36). For zinc, we used recommendations issued by the International Zinc Nutrition Consultative Group, considering 25% zinc absorption for women with an unrefined, cereal-based diet (37).

### Statistical analysis

Data management and statistical analysis were performed in Stata (version 14.2; StataCorp). Baseline characteristics of study participants were summarized by study group as means ± SDs for continuous variables and as percentages for categorical variables. Linear regression models were fitted on the crude observed intake values to compare energy and nutrient intakes between the control and intervention group (median and IQR for values with a skewed distribution or mean ± SD for normally distributed values), adjusted for health center to account for the study design and interviewer as fixed-effect covariates (38). The assumption of normality was checked by visual inspection of histograms and QQ-plots of the residuals. Crude values were used for normally distributed outcomes (% of energy from protein and % of energy from carbohydrates), and outcome variables were log-transformed when these assumptions were violated to test the significance of differences between study groups, using the control as a reference. For outcomes that were not amenable to transformation (% of energy from fat, vitamin B-12, and vitamin C), we applied quantile regression to estimate median differences between study groups. We carried out sensitivity analysis by excluding potentially influential outliers with standardized residuals >|3|. A 2-sided significance level of *P* < 0.05 was applied for all analyses.

## Results

### Characteristics of participants

A total of 470 recalls (including 36 women from the back-up list) were completed: 253 women in the control group and 217 in the intervention arm. The mean duration of supplement intake was 117 d for the control group (IFA) and 122 d (IFA+BEP) for the intervention group at the time of the recall. Study arms were well balanced in terms of sociodemographic and maternal characteristics at the time of interview, while slightly more women in the intervention group were underweight at the time of enrollment in the RCT (**Table 2**).

**TABLE 2:** Characteristics of pregnant women, by study arm^1^

	Control (IFA) (*n* = 253)	Intervention (IFA+BEP) (*n* = 217)
Age,^2^ y	25.5 ± 6.30	25.4 ± 6.63
Ethnicity^2^		
Bwaba	146 (57.7)	125 (57.6)
Mossi	91 (36.0)	71 (32.7)
Other	16 (6.32)	19 (8.76)
Education^2^		
None	127 (50.2)	93 (42.9)
Primary	107 (42.3)	97 (44.7)
Secondary	19 (7.51)	25 (11.5)
Gravidity^2^		
0	48 (19.0)	49 (22.6)
1–2	92 (36.4)	62 (28.6)
≥3	113 (44.7)	106 (48.9)
Gestational age, wk	27.5 ± 6.65	27.5 ± 6.86
Trimester		
First	1 (0.40)	0 (0.00)
Second	123 (48.6)	104 (47.9)
Third	129 (51.0)	113 (52.1)
Weight,^2^ kg	59.0 ± 7.93	59.0 ± 9.53
BMI,^2^ kg/m^2^	22.3 ± 2.79	22.2 ± 3.15
BMI category^2^		
Underweight, <18.5 kg/m^2^	10 (3.95)	20 (9.22)
Normal, 18.5–24.9 kg/m^2^	212 (83.8)	165 (76.0)
Overweight, 25–29.9 kg/m^2^	28 (11.1)	27 (12.4)
Obese, ≥30 kg/m^2^	3 (1.19)	5 (2.30)
MUAC,^2^ mm	265 ± 25.5	263 ± 28.2
No. of meals during recall day	4.06 ± 0.86	4.05 ± 0.86
Quantity of food intake during recall day		
Less than usual	32 (12.6)	24 (11.1)
As much as usual	194 (76.7)	163 (75.1)
More than usual	27 (10.7)	30 (13.8)
Variety of food intake during recall day		
Less than usual	13 (5.1)	11 (5.1)
As usual	205 (81.0)	170 (78.3)
More than usual	35 (13.8)	36 (16.6)
Special circumstances during recall day		
No appetite	4 (1.58)	3 (1.38)
Sick	5 (1.98)	4 (1.84)
Family visit/funeral	3 (1.19)	1 (0.46)
BEP intake		
Not consumed	251 (99.2)	5 (2.30)
One-half portion consumed	0 (0.00)	4 (1.84)
Three-fourths portion consumed	0 (0.00)	8 (3.69)
Full portion consumed	2 (0.80)	200 (92.2)
IFA intake	252 (99.6)	215 (99.1)

1 Values are means ± SDs or frequencies (%). BEP, balanced energy-protein; IFA, iron–folic acid; MISAME, MIcronutriments pour la SAnté de la Mère et de l'Enfant; MUAC, midupper arm circumference.

2 Baseline data were collected at inclusion in the MISAME-III trial.

### Food-consumption pattern and supplement intake

Twelve percent of women (*n* = 56) reported having eaten less than usual, 76% (*n* = 357) reported having eaten as usual, and 12% (*n* = 57) reported eating more than usual on the day of the 24HR. Furthermore, women indicated that their food variety was less than usual in 5% of the recalls (*n* = 57), as usual in 80% (*n* = 376), and more varied than usual in 15% of the recalls (*n* = 71) (Table 2).

**FIGURE 1:**
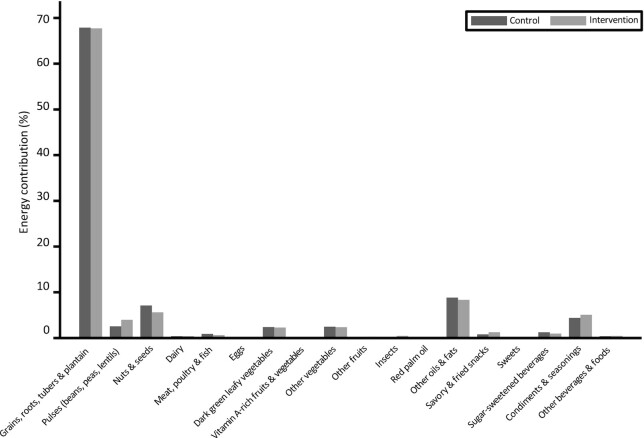
Energy contribution per food group of the base diet, according to classification of the MDD-W indicator, by control and intervention group. MDD-W, Minimum Dietary Diversity for Women.

**Figure 1** shows the contribution (in %) of each food group of the Minimum Dietary Diversity for Women (MDD-W) classification including the optional categories (39) to total energy intake of the base diet (BEP excluded), by study arm. The results show that the base diet is mainly cereal based. Almost all women (95%) consumed the main staple dish *tô*, with a mean ± SD portion size of 543  ± 212 g and a corresponding 42.3 ± 23.3% contribution to total energy intake. *Tô* is a stiff cereal dough often served with a watery sauce containing green-leafy vegetables (okra, hibiscus, and baobab leaves) or other vegetables such as eggplant, with or without meat, fish, or caterpillars. Other nutritious food groups such as fruit, dairy, eggs, fish, and meat contribute very small amounts to the total energy intake. No large differences or substitution of food groups were observed (Figure 1). Specifically, no meaningful difference was found in the base diet in the intake of sentinel food items, such as peanuts as a snack or dishes that contained peanuts between the intervention and control groups (**Supplemental Table 1**).

Among women in the intervention group, 92% (*n* = 200) consumed the complete portion of the fortified BEP supplement (72 g) the day prior to the assessment. Twelve women indicated that they consumed a part of it (0.5 or 0.75 portion) and 5 women did not consume the BEP supplement at all. Reasons for not consuming the BEP supplement included not feeling well, not feeling hungry, or not receiving the visit from the trial community support staff who provide the daily supplement. Two women from the control group indicated having consumed the BEP supplement. One woman in the control group and 5 women in the intervention group did not take the IFA tablet. No women reported the consumption of any other type of nutritional supplement.

### Energy and nutrient intakes

Median (IQR) energy intake on the recall day in the control group was 1942 (1575, 2405) kcal/d and 2329 (1855, 3008) kcal/d in the intervention group, including supplements (**Table 3**). Women in the intervention group had significantly higher intakes of energy and macro- and micronutrients. Comparing the base diet (without supplementation) between both groups indicates that almost all additional energy and macro- and micronutrients in the intervention group can be attributed to the consumption of the BEP supplement (**Table 4**). No differences were found between trimesters. Sensitivity analyses excluding outliers (*n* = 10) did not change our findings (results not shown). Low energy intakes (minimum 420 kcal/d) were observed in women who reported sickness, lack of appetite, or the consumption of rather nutrient-poor sauces with limited quantities of fresh green leaves and lots of added water. In contrast, high energy intakes (maximum 5057 kcal/d) were observed in women who reported large portions of staple foods, peanuts, beans, legumes, shea butter, or oil, and high micronutrient contents were identified for diets that included sauces containing large amounts of nutrient-dense dried hibiscus/green leaves.

**TABLE 3:** Energy and nutrient intakes and adequacy ratios of diets including supplements, between control and intervention groups^1^

	Control (diet including IFA) (*n* = 253)	Intervention (diet including IFA+BEP) (*n* = 217)	Difference (95% CI)^2^	*P*	NAR control (diet including IFA) (*n* = 253)	NAR intervention (diet including IFA+BEP) (*n* = 217)
Energy, kcal/d	1942 (1575, 2405)	2329 (1855, 3008)	448 (291, 605)	<0.01^3^	NA	NA
Fat, g/d	33.9 (18.4, 57.6)	58.4 (42.1, 81.3)	25.6 (19.1, 32.0)	<0.01^3^	NA	NA
Percentage of energy from fat	16.2 (9.24, 23.4)	23.2 (18.8, 27.9)	6.74 (5.14, 8.34)	<0.01^4^	NA	NA
Protein, g/d	50.6 (37.8, 67.9)	63.7 (49.6, 85.1)	15.2 (9.91, 20.5)	<0.01^3^	0.98 (0.74, 1.00)	1.00 (1.00, 1.00)
Percentage of energy from protein, mean ± SD	10.8 ± 2.43	11.4 ± 1.94	0.51 (0.10, 0.91)	0.01^5^	NA	NA
CHO, g/d	340 (269, 424)	362 (280, 469)	37.5 (12.9, 62.0)	<0.01^3^	1.00 (1.00, 1.00)	1.00 (1.00, 1.00)
Percentage of energy from CHO, mean ± SD	69.8 ± 9.80	62.7 ± 7.41	−7.15 (−8.76, −5.54)	<0.01^5^	NA	NA
Calcium, mg/d	407 (273, 587)	899 (744, 1149)	515 (449, 582)	<0.01^3^	0.50 (0.33, 0.71)	1.00 (0.90, 1.00)
Iron, mg/d	81.9 (76.3, 90.6)	103 (97.1, 110)	20.5 (18.0, 23.0)	<0.01^3^	0.74 (0.69, 0.82)^6^	0.93 (0.87, 1.00)^6^
					1.00 (1.00, 1.00)^6^	1.00 (1.00, 1.00)^6^
					1.00 (1.00, 1.00)^6^	1.00 (1.00, 1.00)^6^
Zinc, mg/d	8.96 (6.74, 11.4)	23.7 (21.4, 26.9)	14.6 (13.7, 15.5)	<0.01^3^	0.57 (0.43, 0.71)^7^	1.00 (1.00, 1.00)^7^
Vitamin A, RAE/d	154 (75.7, 379)	910 (824, 1133)	764 (656, 873)	<0.01^3^	0.29 (0.14, 0.71)	1.00 (1.00, 1.00)
Thiamin, mg/d	0.68 (0.47, 1.07)	2.13 (1.88, 2.49)	1.40 (1.29, 1.51)	<0.01^3^	0.57 (0.39, 0.89)	1.00 (1.00, 1.00)
Riboflavin, mg/d	0.64 (0.46, 0.91)	1.99 (1.79, 2.32)	1.33 (1.24, 1.42)	<0.01^3^	0.53 (0.38, 0.76)	1.00 (1.00, 1.00)
Niacin, mg/d	7.30 (5.48, 9.90)	22.3 (20.1, 24.9)	14.8 (13.8, 15.9)	<0.01^3^	0.52 (0.39, 0.71)	1.00 (1.00, 1.00)
Vitamin B-6, mg/d	1.03 (0.80, 1.40)	2.90 (2.65, 3.28)	1.85 (1.75, 1.95)	<0.01^3^	0.64 (0.50, 0.88)	1.00 (1.00, 1.00)
Folate, μg/d	605 (544, 754)	1006 (931, 1156)	399 (353, 444)	<0.01^3^	1.00 (1.00, 1.00)	1.00 (1.00, 1.00)
Vitamin B-12, mg/d	0.05 (0.01, 0.25)	2.63 (2.61, 2.79)	2.33 (2.02, 2.63)	<0.01^4^	0.02 (0.00, 0.11)	1.00 (1.00, 1.00)
Vitamin C, mg/d	35.2 (19.0, 53.8)	133 (115, 156)	102 (91.6, 113)	<0.01^4^	0.50 (0.28, 0.77)	1.00 (1.00, 1.00)

1 Values are medians (IQR) unless otherwise indicated. BEP, balanced energy-protein; CHO, carbohydrate; IFA, iron–folic acid; NA, not applicable; NAR, Nutrient Adequacy Ratio; RAE, retinol activity equivalents.

2 Group differences were estimated using crude observed nutrient intake values by fitting linear regression models adjusted for health center and enumerator as fixed-effect covariates.

3 Significant differences between study groups were tested on log-transformed values in case the assumption of normality was violated.

4 Significant differences between study groups were tested by fitting quantile regression models based on the crude values in case the assumption of normality was violated but the outcomes were not amenable to transformation.

5 Significant differences between study groups were tested on crude values in case the data were normally distributed.

6 Values, from top to bottom, are adjusted for low (5%), medium (10%), and high (16%) bioavailability for iron.

7 Values are adjusted for 25% bioavailability for zinc in our setting of predominantly cereal-based diets.

**TABLE 4:** Energy and nutrient intakes and adequacy ratios of base diets, between control and intervention groups^1^

	Control (base diet) (*n* = 253)	Intervention (base diet) (*n* = 217)	Difference (95% CI)^2^	*P*	NAR control (base diet) (*n* = 253)	NAR intervention (base diet) (*n* = 217)
Energy, kcal/d	1942 (1575, 2405)	1936 (1480, 2615)	70.8 (-86.0, 227)	0.47^3^	NA	NA
Fat, g/d	33.9 (18.4, 57.6)	33.4 (17.1, 55.6)	0.63 (−5.83, 7.09)	0.97^3^	NA	NA
Percentage of energy from fat	16.2 (9.24, 23.4)	15.1 (8.76, 21.9)	−0.71 (−2.47, 1.05)	0.63^4^	NA	NA
Protein, g/d	50.6 (37.8, 67.9)	49.3 (36.0, 72.2)	1.30 (−3.97, 6.58)	0.76^3^	0.98 (0.74, 1.00)	0.95 (0.73, 1.00)
Percentage of energy from protein, mean ± SD	10.8 ± 2.43	10.7 ± 2.26	−0.18 (-0.61, 0.25)	0.42^5^	NA	NA
CHO, g/d	340 (269, 424)	338 (257, 447)	15.12 (−9.44, 36.7)	0.23^3^	1.00 (1.00, 1.00)	1.00 (1.00, 1.00)
Percentage of energy from CHO, mean ± SD	69.8 ± 9.80	70.8 ± 9.46	0.92 (−0.84, 2.69)	0.31^5^	NA	NA
Calcium, mg/d	407 (273, 587)	409 (277, 664)	35.5 (−29.6, 101)	0.61^3^	0.50 (0.33, 0.71)	0.50 (0.34, 0.80)
Iron, mg/d	16.9 (11.4, 25.7)	16.8 (11.1, 23.3)	−0.37 (−2.64, 1.90)	0.41^3^	0.15 (0.10, 0.23)^6^	0.15 (0.10, 0.21)^6^
					0.30 (0.20, 0.46)^6^	0.30 (0.20, 0.41)^6^
					0.50 (0.34, 0.76)^6^	0.49 (0.32, 0.68)^6^
Zinc, mg/d	8.96 (6.74, 11.4)	8.94 (6.56, 11.9)	0.18 (−0.63, 1.01)	0.68^3^	0.57 (0.43, 0.71)^7^	0.56 (0.42, 0.76)^7^
Vitamin A, RAE/d	154 (75.7, 379)	148 (65.2, 373)	25.7 (−81.3, 132)	0.65^3^	0.29 (0.14, 0.71)	0.28 (0.12, 0.70)
Thiamin, mg/d	0.68 (0.47, 1.07)	0.75 (0.50, 1.10)	0.06 (−0.05, 0.16)	0.17^3^	0.57 (0.39, 0.89)	0.62 (0.41, 0.91)
Riboflavin, mg/d	0.64 (0.46, 0.91)	0.60 (0.46, 0.92)	−0.16 (-0.10, 0.07)	0.45^3^	0.53 (0.38, 0.76)	0.50 (0.34, 0.76)
Niacin, mg/d	7.30 (5.48, 9.90)	7.39 (5.24, 9.90)	0.42 (−0.57, 1.42)	0.63^3^	0.52 (0.39, 0.71)	0.53 (0.37, 0.71)
Vitamin B-6, mg/d	1.03 (0.80, 1.40)	1.05 (0.80, 1.40)	0.03 (−0.06, 0.12)	0.72^3^	0.64 (0.50, 0.88)	0.66 (0.50, 0.88)
Folate, μg/d	205 (144, 354)	209 (145, 356)	16.6 (−27.1, 60.3)	0.76^3^	0.39 (0.28, 0.68)	0.40 (0.28, 0.68)
Vitamin B-12, mg/d	0.05 (0.01, 0.25)	0.04 (0.01, 0.19)	−0.17 (−0.47, 0.13)	0.70^4^	0.02 (0.00, 0.11)	0.02 (0.00, 0.09)
Vitamin C, mg/d	35.2 (19.0, 53.8)	34.1 (18.0, 57.4)	6.47 (−4.16, 17.1)	0.88^4^	0.50 (0.28, 0.77)	0.50 (0.26, 0.85)

1 Values are medians (IQR) unless otherwise indicated. BEP, balanced energy-protein; CHO, carbohydrate; IFA, iron–folic acid; NAR, Nutrient Adequacy Ratio; RAE, retinol activity equivalents.

2 Group differences were estimated using crude observed nutrient intake values by fitting linear regression models adjusted for health center and enumerator as fixed-effect covariates.

3 Significant differences between study groups were tested on log-transformed values in case the assumption of normality was violated.

4 Significant differences betewen study groups were tested by fitting quantile regression models based on the crude values in case the assumption of normality was violated but the outcomes were not amenable to transformation.

5 Significant differences between study groups were tested on crude values in case the data were normally distributed.

6 Values, from top to bottom, are adjusted for low (5%), medium (10%), and high (16%) bioavailability for iron.

7 Values are adjusted for 25% bioavailability for zinc in our setting of predominantly cereal-based diets.

### Nutrient adequacy

When omitting supplement intake (IFA or BEP) from the analysis, NARs from women in the control (IFA) and intervention (IFA+BEP) groups were low for zinc, calcium, thiamin, riboflavin, niacin, vitamin B-6, and vitamin C and very low for iron, vitamin A, folate, and vitamin B-12 (Table 4). The EARs for folate and iron, assuming medium (10%) and high (16%) bioavailability, were met in the control group by providing standard daily IFA tablets (Table 3). It is, however, likely that the bioavailability of iron is low (5%) in our setting due to a primarily plant-based diet (36). In the intervention group, providing the BEP supplement in addition to the base diet increased the adequacy ratio for all nutrients to at least the level of the respective EAR (Table 3).

## Discussion

We report that the distribution of BEP supplements led to significantly higher intakes for energy and macro- and micronutrients as compared with women from the control group. Our results indicate that in case the full portion of BEP was consumed there was little to no displacement of the food intake among pregnant women in Burkina Faso. In fact, nutrient intakes in the base diet were remarkably similar across study groups, and all micronutrient intakes were inadequate relative to the EAR. The BEP supplement filled all the nutrient gaps and was able to account for the additional energy requirement during pregnancy, making it a valuable contribution to the diet of undernourished pregnant women.

For 13 supplementation studies that reported small to no impact on birth outcomes, authors have speculated that the supplement might have displaced at least part of the usual diet (19, 20). Therefore, it is essential that food-supplementation trials are accompanied by well-conducted dietary intake studies to assess possible substitution effects to aid in the assessment of the trial's outcomes. To our knowledge, this is one of the few studies that investigated the displacement of food by BEP supplementation in pregnant women in low- and middle-income countries. In the MISAME-II trial, a locally produced LNS based on soy flour, peanut butter, oil, and sugar with a premix of multiple micronutrients was compared with an MMN tablet. In this study, the LNS appeared to be an extra allowance for women during pregnancy (28). The few other studies on maternal food supplementation date from more than 30 to 40 y ago (28, 40). Other peer-reviewed literature focuses on dietary intake differences between study arms in supplementation trials in children aged 3 to 60 mo. The general conclusion of trials in Burkina Faso (23), Malawi (41–43), Ghana (44), Uganda (45), Honduras (46), and Bangladesh (47, 48) is that LNSs improved energy and macro- and micronutrient intakes without displacing other foods in the diet of young children. For supplements based on cereals (e.g., corn soy blend), some level of substitution was found (23, 42, 43).

Without considering the BEP supplements, pregnant women's nutrient intakes were inadequate. The nutrient inadequacies reported in this study are comparable to other countries in Africa. A systematic review from 20 studies in resource-poor settings in sub-Saharan Africa reported inadequate micronutrient intakes of pregnant women for vitamin C, niacin, vitamin B-6, folate, vitamin B-12, iron, and zinc (49). In Mali, the NARs for vitamin A and calcium were also below recommended levels in 70–80% of households (50); and in Niger, usual dietary intakes of vitamin A, thiamin, riboflavin, niacin, folate, and vitamin C were inadequate for >50% of pregnant and lactating women with calcium and vitamin B-12 intakes inadequate for all women, comparable to our study (51). These diets typically show imbalances in macronutrients, inadequate micronutrient levels, and are predominantly plant-based (52), which is most likely the cause of inadequate iron and zinc intakes (53).

Addressing the nutritional needs of pregnant and lactating women is key to reach the UN 2030 Sustainable Development Goals (54). In addition to food-based programs, our results show that fortified food supplementation to improve nutrient intakes can be a promising strategy. The mean energy intake of 1942 kcal/d in the control group does not seem to address the increased daily energy requirement in the second (290 kcal) and third (465 kcal) trimester of pregnancy, considering an average energy requirement of 2360 kcal/d for nonpregnant women aged 18–30 y with a physical activity level of 1.75 (i.e., “moderately active”) and mean weight of 59 kg (2, 55). BEP supplementation could thus play a crucial role in addressing increasing energy and nutrient requirement throughout pregnancy.

Strengths of this study include the detailed semi-structured 24HR collection tool, in which almost all commonly consumed foods in Burkina Faso could be selected from a drop-down menu and included questions on type or preparation method. This way, a detailed list of food items was collected using a standardized 5-pass method for all participants. Instead of using mean or standard recipe data, we collected information on all individual ingredients and the preparation method of mixed dishes to improve the quality of our findings. We thereby not only capture potential variations across groups due to differences in portion sizes, but such data also allows us to capture potential differences due to displacement in the type of ingredients used for cooking.

Our study has some limitations. First, the 24HR method is inherently prone to measurement error (29). While this is an inevitable limitation, our goal was to compare differences between the 2 groups and estimate population average dietary intakes. Any possible systematic error of estimation might therefore be present in both study arms as women were randomly allocated to the study groups and it is unlikely that there would be any different behavior in under- or overreporting. With intensive training, close supervision, recalls on all days of the week, a standardized multiple-pass interview technique, and the use of multiple measurement aids (e.g., photos, clay, salted replicas) specifically designed for the study area allowing for visual and individual portion-size estimations, we limited potential errors as much as possible (56). Second, since the main study objective was a comparison between group means of intakes, we performed only 1 recall per woman. With this information, we were able to calculate adequacy ratios at a population level but are unable to calculate individual level (mean) probability of adequacy across nutrients in this trial due to a lack of estimates of the intra-person or day-to-day variance of the observed nutrient intakes. Values from other studies may vary from our study population and incorrect use may influence the accuracy of prevalence estimates (57). Additionally, we cannot entirely rule out group imbalances in unmeasured prognostic factors of dietary intake (e.g., physical activity level or basic metabolic rate). Any influence from the IFA tables on the diet—for example, due to nausea—also cannot be ruled out. Third, analyses were limited to available information in the West African FCT and additional tables from Burkina Faso. Even though the West African FCT was updated in 2019, not all locally consumed foods could be found in the database. Consensus was reached between the researchers on which alternative food items to use.

In conclusion, our study results show that BEP supplements improved nutrient intakes of pregnant women without displacing foods that are part of the base diet. Findings of the MISAME-III efficacy trial are critical to determine if the observed positive dietary contribution also leads to improved birth outcomes and infant growth.

## Supplementary Material

nxab289_Supplemental_Files

## Data Availability

Anonymized individual participant data will be available at https://biblio.ugent.be/.

## Notes

This work was supported by the Bill & Melinda Gates Foundation (OPP1175213). Under the grant conditions of the Foundation, a Creative Commons Attribution 4.0 Generic License has already been assigned to the Author Accepted Manuscript version that might arise from this submission.

Author disclosures: The authors report no conflicts of interest.

Supplemental Table 1 is available from the “Supplementary data” link in the online posting of the article and from the same link in the online table of contents available on https://academic.oup.com/jn/.

Abbreviations used: BEP, balanced energy-protein; EAR, Estimated Average Requirement; FCT, food-composition table; IFA, iron–folic acid; LNS, lipid-based nutrient supplement; MISAME, MIcronutriments pour la SAnté de la Mère et de l'Enfant; MMN, multiple-micronutrient; NAR, Nutrient Adequacy Ratio; RCT, randomized controlled trial; SELEVER, Soutenir l'Exploitation familiale pour Lancer l’Élevage des Volailles et Valoriser l’Économie Rurale; 24HR, 24-h recall.

## References

[1] Williamson CS . Nutrition in pregnancy. Nutr Bull. 2006;31:28–59.

[2] Butte NF , King JC. Energy requirements during pregnancy and lactation. Public Health Nutr. 2005;8:1010–27. 16277817 10.1079/phn2005793

[3] Bourassa MW , Osendarp SJM, Adu-Afarwuah S, Ahmed S, Ajello C, Bergeron G, Black R, Christian P, Cousens S, de Pee S et al. Review of the evidence regarding the use of antenatal multiple micronutrient supplementation in low- and middle-income countries. Ann N Y Acad Sci. 2019;1444:6–21. 31134643 10.1111/nyas.14121 PMC6852202

[4] Mousa A , Naqash A, Lim S. Macronutrient and micronutrient intake during pregnancy: an overview of recent evidence. Nutrients. 2019;11:1–20. 10.3390/nu11020443 PMC6413112 30791647

[5] Gernand AD , Schulze KJ, Stewart CP, West KP Jr, Christian P. Micronutrient deficiencies in pregnancy worldwide: health effects and prevention. Nat Rev Endocrinol. 2016;12:274. 27032981 10.1038/nrendo.2016.37 PMC4927329

[6] Ramakrishnan U , Imhoff-Kunsch B, Martorell R. Maternal nutrition interventions to improve maternal, newborn, and child health outcomes. Nestle Nutr Inst Workshop Ser. 2014;78:71–80. 24504208 10.1159/000354942

[7] Ramakrishnan U . Prevalence of micronutrient malnutrition worldwide. Nutr Rev. 2002;60:S46–52. 12035858 10.1301/00296640260130731

[8] Huybregts LF , Roberfroid DA, Kolsteren PW, Van Camp JH. Dietary behaviour, food and nutrient intake of pregnant women in a rural community in Burkina Faso. Matern Child Nutr. 2009;5:211–22. 20572925 10.1111/j.1740-8709.2008.00180.x PMC6860675

[9] Arsenault JE , Nikiema L, Allemand P, Ayassou KA, Lanou H, Moursi M, De Moura FF, Martin-Prevel Y. Seasonal differences in food and nutrient intakes among young children and their mothers in rural Burkina Faso. J Nutr Sci. 2014;3:1–9. 10.1017/jns.2014.53 PMC4473133 26101623

[10] Martin-Prevel Y , Allemand P, Nikiema L, Ayassou KA, Ouedraogo HG, Moursi M, De Moura FF. Biological status and dietary intakes of iron, zinc and vitamin A among women and preschool children in rural Burkina Faso. PLoS One. 2016;11:e0146810. 26991908 10.1371/journal.pone.0146810 PMC4798773

[11] Becquey E , Martin-Prevel Y. Micronutrient adequacy of women's diet in urban Burkina Faso is low. J Nutr. 2010;140:2079S–85S. 20881079 10.3945/jn.110.123356

[12] Becquey E , Delpeuch F, Konaté AM, Delsol H, Lange M, Zoungrana M, Martin-Prevel Y. Seasonality of the dietary dimension of household food security in urban Burkina Faso. Br J Nutr. 2012;107:1860–70. 22017887 10.1017/S0007114511005071

[13] Arimond M , Vitta BS, Martin-Prével Y, Moursi M, Dewey KG. Local foods can meet micronutrient needs for women in urban Burkina Faso, but only if rarely consumed micronutrient-dense foods are included in daily diets: a linear programming exercise. Matern Child Nutr. 2017;14:e12461. 10.1111/mcn.12461 PMC6866244 28464499

[14] Tran NT , Nguyen LT, Berde Y, Low YL, Tey SL, Huynh DTT. Maternal nutritional adequacy and gestational weight gain and their associations with birth outcomes among Vietnamese women. BMC Pregnancy Childbirth. 2019;19:1–10. 31801514 10.1186/s12884-019-2643-6 PMC6894140

[15] Lassi ZS , Padhani ZA, Rabbani A, Rind F, Salam RA, Das JK, Bhutta ZA. Impact of dietary interventions during pregnancy on maternal, neonatal, and child outcomes in low- and middle-income countries. Nutrients. 2020;12:531–47. 10.3390/nu12020531 PMC7071393 32092933

[16] Keats EC , Das JK, Salam RA, Lassi ZS, Imdad A, Black RE, Bhutta ZA. Effective interventions to address maternal and child malnutrition: an update of the evidence. Lancet Child Adolesc Heal. 2021;4642:1–18. 10.1016/S2352-4642(20)30274-1 33691083

[17] WHO . WHO recommendations on antenatal care for a positive pregnancy experience. [Internet]. 2016. Available from: https://www.who.int/publications/i/item/9789241549912. 28079998

[18] Stevens B , Buettner P, Watt K, Clough A, Brimblecombe J, Judd J. The effect of balanced protein energy supplementation in undernourished pregnant women and child physical growth in low- and middle-income countries: a systematic review and meta-analysis. Matern Child Nutr. 2015;11:415–32. 25857334 10.1111/mcn.12183 PMC6860195

[19] Ota E , Hori H, Mori R, Tobe-Gai R, Farrar D. Antenatal dietary education and supplementation to increase energy and protein intake. Cochrane Database Syst Rev. 2015;6:1–103. 10.1002/14651858.CD000032.pub3 PMC12634316 26031211

[20] Kramer MS , Kakuma R. Energy and protein intake in pregnancy. Cochrane Database Syst Rev. 2003;6:1–80. 10.1002/14651858.CD000032 14583907

[21] Imdad A , Bhutta ZA. Maternal nutrition and birth outcomes: effect of balanced protein-energy supplementation. Paediatr Perinat Epidemiol. 2012;26:178–90. 22742610 10.1111/j.1365-3016.2012.01308.x

[22] Fatima S , Malkova D, Wright C, Gerasimidis K. Impact of therapeutic food compared to oral nutritional supplements on nutritional outcomes in mildly underweight healthy children in a low-medium income society. Clin Nutr. 2018;37:858–63. 28343801 10.1016/j.clnu.2017.03.006

[23] Cliffer IR , Masters WA, Rogers BL. Fortified blended flour supplements displace plain cereals in feeding of young children. Matern Child Nutr. 2020;17:1–14. 10.1111/mcn.13089 PMC7988859 32990388

[24] Lachat C , Hawwash D, Ocké MC, Berg C, Forsum E, Hörnell A, Larsson C, Sonestedt E, Wirfält E, Åkesson A et al. Strengthening the Reporting of Observational Studies in Epidemiology—Nutritional Epidemiology (STROBE-nut): an extension of the STROBE statement. PLoS Med. 2016;13:1–15. 10.1371/journal.pmed.1002036 PMC4896435 27270749

[25] Vanslambrouck K , de Kok B, Toe LC, De Cock N, Ouédraogo M, Dailey-Chwalibóg T, Hanley-Cook G, Ganaba R, Lachat C, Huybregts L et al. Effect of balanced energy-protein supplementation during pregnancy and lactation on birth outcomes and infant growth in rural Burkina Faso: study protocol for a randomised controlled trial. BMJ Open. 2021;11:1–10. 10.1136/bmjopen-2020-038393 PMC7993280 33762226

[26] Jones L , de Kok B, Moore K, de Pee S, Bedford J, Vanslambrouck K, Toe LC, Lachat C, De Cock N, Ouédraogo M et al. Acceptability of 12 fortified balanced energy protein supplements—insights from Burkina Faso. Matern Child Nutr. 2020;17:(1):e13067.doi: 10.1111/mcn.13067 32757351 PMC7729548

[27] de Kok B , Moore K, Jones L, Vanslambrouck K, Toe LC, Ouédraogo M, Ganaba R, de Pee S, Bedford J, Lachat C et al. Home consumption of two fortified balanced energy protein supplements by pregnant women in Burkina Faso. Matern Child Nutr. 2021;17:(3):e13134. doi:10.1111/mcn.13134. 33405368 PMC8189188

[28] Huybregts L . Prevention of intrauterine growth retardation by food supplementation in rural Burkina Faso. [Internet]. Ghent University; 2010. Available from: https://biblio.ugent.be/publication/903510.

[29] Gibson RS , Ferguson EL. An interactive 24-hour recall for assessing the adequacy of iron and zinc intakes in developing countries. [Internet]. Washington (DC): IFPRI and CIAT; 2008. Available from: https://ebrary.ifpri.org/digital/collection/p15738coll2/id/128218.

[30] Diop L , Becquey E, Turowska Z, Huybregts L, Ruel MT, Gelli A. Standard minimum dietary diversity indicators for women or infants and young children are good predictors of adequate micronutrient intakes in 24–59-month-old children and their nonpregnant nonbreastfeeding mothers in rural Burkina Faso. J Nutr. 2021;151:412–22. 33326567 10.1093/jn/nxaa360 PMC7850098

[31] FAO/INFOODS . Food composition table for Western Africa: user guide & condensed food composition table. [Internet]. Rome (Italy); 2019. Available from: http://www.fao.org/documents/card/en/c/ca7779b/.

[32] Becquey E , Capon G, Martin-Prével Y. Dietary diversity as a measure of the micronutrient adequacy of women's diets: results from Ouagadougou, Burkina Faso Site (FANTA project). [Internet]. 2009. Available from: https://www.fantaproject.org/sites/default/files/resources/WDDP_BurkinaFaso_Dec09.pdf.

[33] Anvo PM , Toguyéni A, Okoumou AK, Zoungrana-Kaboré CY, Koumelan EP. Nutritional qualities of edible caterpillars Cirina butyrospermi in southwestern of Burkina Faso. Int J Innov Appl Stud. 2016;18:639–45.

[34] Food and Nutrition Board . USDA table of nutrient retention factors, release 6. Natl Acad Press [Internet]. 2007;18. Available from: https://data.nal.usda.gov/system/files/retn06.pdf.

[35] Institute of Medicine Food and Nutrition Board . Dietary Reference Intakes (DRIs): Estimated Average Requirements. [Internet]. 2011. Available from: https://www.nal.usda.gov/sites/default/files/fnic_uploads/recommended_intakes_individuals.pdf.

[36] Allen LH , Carriquiry AL, Murphy SP. Perspective: proposed harmonized nutrient reference values for populations. Adv Nutr. 2020;11:469–83. 31701998 10.1093/advances/nmz096 PMC7231601

[37] Brown KH , Rivera JA, Bhutta Z, Gibson RS, King JC, Lönnerdal B, Ruel MT, Sandtröm B, Wasantwisut E, Hotz C et al. International Zinc Nutrition Consultative Group (IZiNCG) technical document #1. Assessment of the risk of zinc deficiency in populations and options for its control. Food Nutr Bull. 2004;25:S99–203. 18046856

[38] Slimani N , Ferrari P, Ocké M, Welch A, Boeing H, Van Liere M, Pala V, Amiano P, Lagiou A, Mattisson I et al. Standardization of the 24-hour diet recall calibration method used in the European Prospective Investigation into Cancer and Nutrition (EPIC): general concepts and preliminary results. Eur J Clin Nutr. 2000;54:900–17. 11114689 10.1038/sj.ejcn.1601107

[39] FAO and FHI 360 . Minimum dietary diversity for women: a guide to measurement. [Internet]. Rome (Italy): FAO; 2016. Available from: http://www.fao.org/3/i5486e/i5486e.pdf.

[40] Liberato SC , Singh G, Mulholland K. Literature focusing on contextual factors. Food Nutr Res. 2013;57:20499. 10.3402/fnr.v57i0.20499 PMC3827488 24235913

[41] Hemsworth J , Kumwenda C, Arimond M, Maleta K, Phuka J, Rehman AM, Vosti SA, Ashorn U, Filteau S, Dewey KG et al. Lipid-based nutrient supplements increase energy and macronutrient intakes from complementary food among Malawian infants. J Nutr. 2016;146:326–34. 26740684 10.3945/jn.115.215327

[42] Maleta K , Kuittinen J, Duggan MB, Briend A, Manary M, Wales J, Kulmala T, Ashorn P. Supplementary feeding of underweight, stunted Malawian children with a ready-to-use food. J Pediatr Gastroenterol Nutr. 2004;38:152–8. 14734876 10.1097/00005176-200402000-00010

[43] Thakwalakwa CM , Ashorn P, Phuka JC, Cheung YB, Briend A, Maleta KM. Impact of lipid-based nutrient supplements and corn-soy blend on energy and nutrient intake among moderately underweight 8-18-month-old children participating in a clinical trial. Matern Child Nutr. 2015;11:144–50. 24528807 10.1111/mcn.12105 PMC6860175

[44] Adu-Afarwuah S , Lartey A, Brown KH, Zlotkin S, Briend A, Dewey KG. Randomized comparison of 3 types of micronutrient supplements for home fortification of complementary foods in Ghana: effects on growth and motor development. Am J Clin Nutr. 2007;86:412–20. 17684213 10.1093/ajcn/86.2.412

[45] Ickes SB , Adair LS, Brahe CA, Thirumurthy H, Charles B, Myhre JA, Bentley ME, Ammerman AS. Impact of lipid-based nutrient supplementation (LNS) on children's diet adequacy in Western Uganda. Matern Child Nutr. 2015;11:163–78. 25597415 10.1111/mcn.12164 PMC6860344

[46] Flax VL , Siega-Riz AM, Reinhart GA, Bentley ME. Provision of lipid-based nutrient supplements to Honduran children increases their dietary macro- and micronutrient intake without displacing other foods. Matern Child Nutr. 2015;11:203–13. 25819697 10.1111/mcn.12182 PMC6696916

[47] Campbell RK , Hurley KM, Shamim AA, Shaikh S, Chowdhury ZT, Mehra S, De Pee S, Ahmed T, West KP, Christian P. Effect of complementary food supplementation on breastfeeding and home diet in rural Bangladeshi children. Am J Clin Nutr. 2016;104:1450–8. 27680994 10.3945/ajcn.116.135509 PMC5081719

[48] Campbell RK , Hurley KM, Shamim AA, Shaikh S, Chowdhury ZT, Mehra S, Wu L, Christian P. Complementary food supplements increase dietary nutrient adequacy and do not replace home food consumption in children 6–18 months old in a randomized controlled trial in rural Bangladesh. J Nutr. 2018;148:1484–92. 30184222 10.1093/jn/nxy136

[49] Torheim LE , Ferguson EL, Penrose K, Arimond M. Women in resource-poor settings are at risk of inadequate intakes of multiple micronutrients. J Nutr. 2010;140:2051–8. 10.3945/jn.110.123463 20881075

[50] Torheim LE , Ouattara F, Diarra MM, Thiam FD, Barikmo I, Hatløy A, Oshaug A. Nutrient adequacy and dietary diversity in rural Mali: association and determinants. Eur J Clin Nutr. 2004;58:594–604. 15042127 10.1038/sj.ejcn.1601853

[51] Wessells KR , Young RR, Ferguson EL, Ouédraogo CT, Faye MT, Hess SY. Assessment of dietary intake and nutrient gaps, and development of food-based recommendations, among pregnant and lactating women in Zinder, Niger: an Optifood linear programming analysis. Nutrients. 2019;11:1–23. 10.3390/nu11010072 PMC6357040 30609695

[52] Lee SE , Talegawkar SA, Merialdi M, Caulfield LE. Dietary intakes of women during pregnancy in low- and middle-income countries. Public Health Nutr. 2013;16:1340–53. 23046556 10.1017/S1368980012004417 PMC10271363

[53] Gibson RS , Raboy V, King JC. Implications of phytate in plant-based foods for iron and zinc bioavailability, setting dietary requirements, and formulating programs and policies. Nutr Rev. 2018;76:793–804. 30010865 10.1093/nutrit/nuy028

[54] International Food Policy Research Institute . Global Nutrition Report 2016: from promise to impact: ending malnutrition by 2030. Washington (DC): International Food Policy Research Institute (IFPRI); 2016.

[55] FAO; WHO; UNU . Human energy requirements: report of a Joint FAO/WHO/UNU Expert Consultation: Rome, 17–24 October 2001. [Internet]. 2001. Available from: http://www.fao.org/3/y5686e/y5686e08.htm.

[56] Gibson RS , Ruth Charrondiere U, Bell W. Measurement errors in dietary assessment using self-reported 24-hour recalls in low-income countries and strategies for their prevention. Adv Nutr. 2017;8:980–91. 29141979 10.3945/an.117.016980 PMC5683000

[57] French CD , Arsenault JE, Arnold CD, Haile D, Luo H, Dodd KW, Vosti SA, Slupsky CM, Engle-Stone R. Within-person variation in nutrient intakes across populations and settings: implications for the use of external estimates in modeling usual nutrient intake distributions. Adv Nutr. 2021;12:429–51. 33063105 10.1093/advances/nmaa114 PMC8262514

