# Supplementary material for: Fortified Balanced Energy-Protein Supplements Increase Nutrient Adequacy without Displacing Food Intake in Pregnant Women in Rural Burkina Faso
Source: J Nutr. 2021 Sep 7;151(12):3831–40. doi: 10.1093/jn/nxab289 (PMC8643591; doi:10.1093/jn/nxab289)
Supplement: nxab289_Supplemental_Files [file nxab289_supplemental_files.zip › Manuscript BEP supplements_Figure1_revision.pptx]

## Slide 1
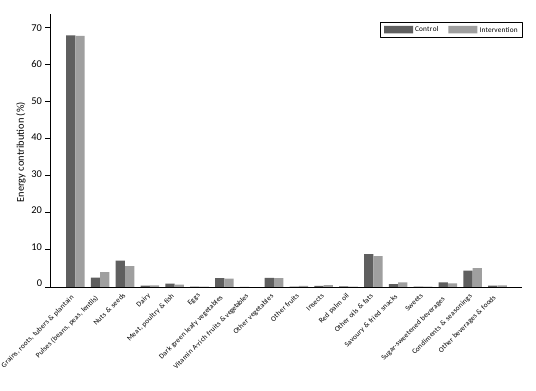

Intervention
Control
70
60
50
40
30
20
10
0
Energy contribution (%)
Eggs
Dairy
Insects
Sweets
Other fruits
Red palm oil
Nuts & seeds
Other oils & fats
Other vegetables
Meat, poultry & fish
Savoury & fried snacks
Other beverages & foods
Condiments & seasonings
Pulses (beans, peas, lentils)
Dark green leafy vegetables
Sugar-sweetened beverages
Grains, roots, tubers & plantain
Vitamin A-rich fruits & vegetables
