# Supplementary material for: Fortified Balanced Energy-Protein Supplements Increase Nutrient Adequacy without Displacing Food Intake in Pregnant Women in Rural Burkina Faso
Source: J Nutr. 2021 Sep 7;151(12):3831–40. doi: 10.1093/jn/nxab289 (PMC8643591; doi:10.1093/jn/nxab289)
Supplement: nxab289_Supplemental_Files [file nxab289_supplemental_files.zip › Manuscript BEP supplements_Supplementary table1.docx]

**Title:** Fortified Balanced Energy-Protein Supplements Increase Nutrient Adequacy without Displacing Food Intake in Pregnant Women in Rural Burkina Faso

**First author:** Brenda de Kok, MSc.

**Document type:** Online Supplementary Material

**Supplementary table 1. Percent energy contribution by food groups and important food items within group^1^**

|  | Control (IFA)  *n* = 253 | Intervention (IFA + BEP)  *n* = 217 |
| --- | --- | --- |
| Grains, white roots, tubers, and plantains | 68.0 | 68.0 |
| Maize | 53.5 | 53.2 |
| Rice | 8.88 | 9.61 |
| Beignet | 1.61 | 1.22 |
| Bread | 1.16 | 0.91 |
| Pasta | 0.78 | 0.36 |
| Sorghum | 0.97 | 1.30 |
| Cassava | 0.49 | 0.55 |
| Pulses (beans, peas, and lentils) | 2.55 | 3.95 |
| Beans | 1.70 | 2.43 |
| Cowpea | 0.53 | 1.29 |
| Nuts and seeds | 7.15 | 5.61 |
| Peanuts | 7.08 | 5.61 |
| Dairy | 0.39 | 0.39 |
| Meat, poultry, and fish | 0.90 | 0.61 |
| Eggs | 0.11 | 0.07 |
| Dark green leafy vegetables | 2.40 | 2.28 |
| Hibiscus leaves | 0.93 | 0.70 |
| Baobab leaves | 0.62 | 1.23 |
| Wild jute leaves | 0.58 | 0.54 |
| Vitamin A-rich fruits & vegetables | 0.01 | 0.00 |
| Other vegetables | 2.47 | 2.37 |
| Okra | 1.21 | 0.92 |
| Eggplant | 0.58 | 0.73 |
| Other fruits | 0.07 | 0.27 |
| Insects | 0.26 | 0.46 |
| Red palm oil | 0.17 | 0.08 |
| Other oils and fats | 8.83 | 8.52 |
| Vegetable oil | 4.79 | 4.39 |
| Shea butter | 3.29 | 3.09 |
| Peanut oil | 0.74 | 0.86 |
| Savoury and fried snacks | 0.79 | 1.26 |
| Cake | 0.26 | 0.55 |
| Sweets | 0.07 | 0.05 |
| Sugar and sweetened beverages | 1.25 | 0.96 |
| Condiments and seasonings | 4.27 | 4.92 |
| Sugar | 1.91 | 2.00 |
| African locust bean | 0.75 | 0.99 |
| Maggi | 0.70 | 0.66 |
| Other beverages and foods | 0.38 | 0.44 |
| Sorghum beer | 0.51 | 0.36 |
| Coffee | 0.35 | 0.58 |

^1^Food items with at least 0.5% energy contribution were considered as important.
